# MicroRNA-944 Affects Cell Growth by Targeting EPHA7 in Non-Small Cell Lung Cancer

**DOI:** 10.3390/ijms17101493

**Published:** 2016-09-26

**Authors:** Minxia Liu, Kecheng Zhou, Yi Cao

**Affiliations:** 1 Laboratory of Molecular and Experimental Pathology, Kunming Institute of Zoology, Chinese Academy of Sciences, Kunming 650223, China; L-M-X101@163.com (M.L.); zhoukecheng1988@gmail.com (K.Z.); 2 Kunming College of Life Science, University of Chinese Academy of Sciences, Kunming 650223, China

**Keywords:** non-small cell lung cancer (NSCLC), miR-944, regulation, cell proliferation, EPHA7

## Abstract

MicroRNAs (miRNAs) have critical roles in lung tumorigenesis and development. To determine aberrantly expressed miRNAs involved in non-small cell lung cancer (NSCLC) and investigate pathophysiological functions and mechanisms, we firstly carried out small RNA deep sequencing in NSCLC cell lines (EPLC-32M1, A549 and 801D) and a human immortalized cell line 16HBE, we then studied miRNA function by cell proliferation and apoptosis. cDNA microarray, luciferase reporter assay and miRNA transfection were used to investigate interaction between the miRNA and target gene. miR-944 was significantly down-regulated in NSCLC and had many putative targets. Moreover, the forced expression of miR-944 significantly inhibited the proliferation of NSCLC cells in vitro. By integrating mRNA expression data and miR-944-target prediction, we disclosed that EPHA7 was a potential target of miR-944, which was further verified by luciferase reporter assay and microRNA transfection. Our data indicated that miR-944 targets EPHA7 in NSCLC and regulates NSCLC cell proliferation, which may offer a new mechanism underlying the development and progression of NSCLC.

## 1. Introduction

Lung cancer is the leading cause of cancer death worldwide, with increasing incidence and mortality. Among all types of lung cancer, non-small cell lung cancer (NSCLC) is the most common type, occurring in approximately 85% of cases [1]. NSCLC is subdivided into three subtypes: adenocarcinoma (AC), squamous cell carcinoma (SCC), and large cell carcinoma (LCC). Although some progress has been made in clinical and experimental oncology, the prognosis of NSCLC is still dismal and the 5-year survival rate is approximately 15% [2]. Understanding the molecular mechanism of cancer development and progression is crucial for early diagnosis and effective treatment. Therefore, further exploration of the underlying mechanism of NSCLC is still urgently needed to generate more effective diagnosis and treatment of NSCLC. The development and progression of NSCLC was predominately driven by genetic and epigenetic alterations [3]. Recently, accumulating reports have described that microRNAs (miRNAs) are implicated in the initiation and progression of lung cancer [4].

miRNAs, a class of small non-coding RNAs, regulates gene expression by triggering either mRNA degradation or translation repression by binding to the 3′-untranslated region (3′-UTR) of a target mRNA [5]. They are broadly wrapped in various biological processes including cell communication, development and differentiations [5]. Increasing evidence has demonstrated that deregulations of miRNAs are correlated with various human cancers and some miRNAs act as an oncogene or tumor suppressor gene (TSG) in human cancers [6]. Moreover, miRNA is considered to be a promising new class of tumor biomarker or therapeutic target [7]. In NSCLCs, some miRNAs have been identified in the proliferation, invasion, metastasis and chemo-resistance of NSCLC [4]. However, roles and mechanisms of miRNAs remain largely unknown in NSCLCs. Therefore, it was of great value to uncover the functions and mechanisms of miRNAs during the development and progression of NSCLC.

This study aimed to screen out aberrantly expressed miRNAs involved in NSCLC. Firstly, we used deep sequencing to characterize miRNA profiles in cultured NSCLC cells. Subsequently, through bioinformatic analysis, miR-944 was chosen for further investigation of its pathophysiological function and molecular mechanism in NSCLCs.

## 2. Results

### 2.1. The Cancer-Related miRNAs Were Screened in Cultured NSCLC Cells

**Figure 1:**
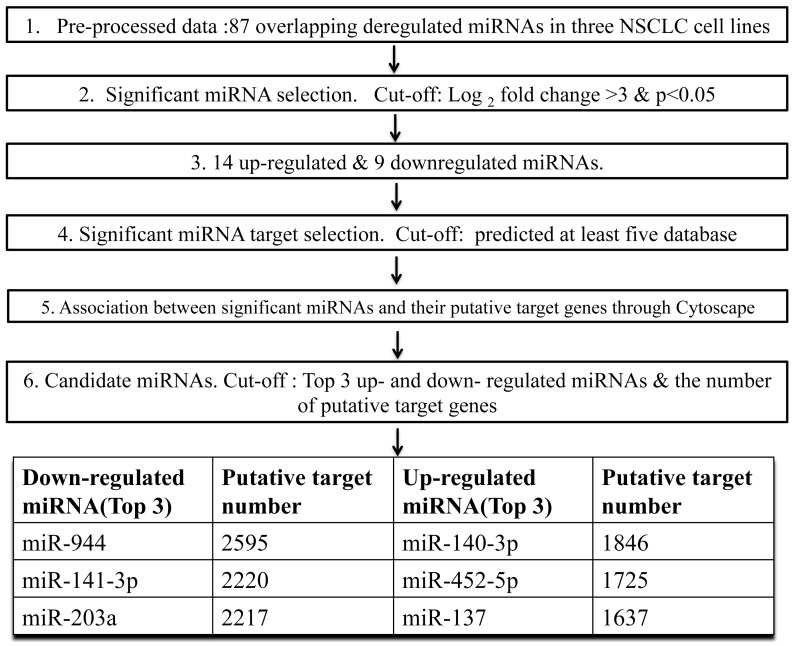
Research strategy of miR-944 screening in NSCLC cell lines.

To identify potential miRNAs involved in lung carcinogenesis, we determine the miRNA expression profile in three human NSCLC cell lines representing different types of NSCLC (EPLC-32M1, SCC; A549, AC; 801D, LCC) and an immortalized human bronchial epithelial cell line (16HBE) through small RNA deep sequencing. The differentially expressed miRNAs were listed in Additional file 1. Compared with 16HBE, 87 consensus miRNAs were significantly deregulated in three NSCLC cell lines with a fold change (FC) > 2. Meanwhile, 41 miRNAs and 23 miRNAs were altered by more than 4 and 8 folds, respectively. To identify the most significant candidates, miRNAs altered by at least 8 folds (Log_2_(Fold change) > 3) in all three NSCLC cell lines were selected for further study. The research strategy is shown in Figure 1. Under these strict criteria, the 23 miRNAs (14 up-regulated and nine down-regulated miRNAs) were resolved from the sequencing results (Table 1). Interestingly, five of the 23 miRNA genes were localized on chromosome 17 (Table 1). According to the distribution of miRNA genes, a cluster of three miRNAs including miR-10a-3p, miR-10a-5p and miR-10b-5p, were mapped to chromosome 17q21.32. These indicated that these frequently altered miRNAs might result from an abnormality of chromosome 17.

miRwalk and eight prediction programs, including Diana-microT, miRanda, miRDB, RNAbybrid, PICTAR4/5, PITA, RNA22 and TargetScan, were used to predict targets of the selected 23 differentially expressed miRNAs. Potential targets were chosen with positive prediction from at least five databases. To further investigate the associations between these altered miRNAs and their targets, both of them were uploaded to Cytoscape to generate a bipartite miRNA/mRNA regulatory network. Regarding the number of target mRNAs; miR-944, miR-141-3p, and miR-203a ranked top three among down-regulated miRNAs, while miR-140-3p, miR-452-5p, and miR-137 located top three of up-regulated miRNAs (Figure 1). Here, we chose miR-944 for further research.

### 2.2. The miR-944 Expression Was Down-Regulated in NSCLC Tissues and Cell Lines

**Figure 2:**
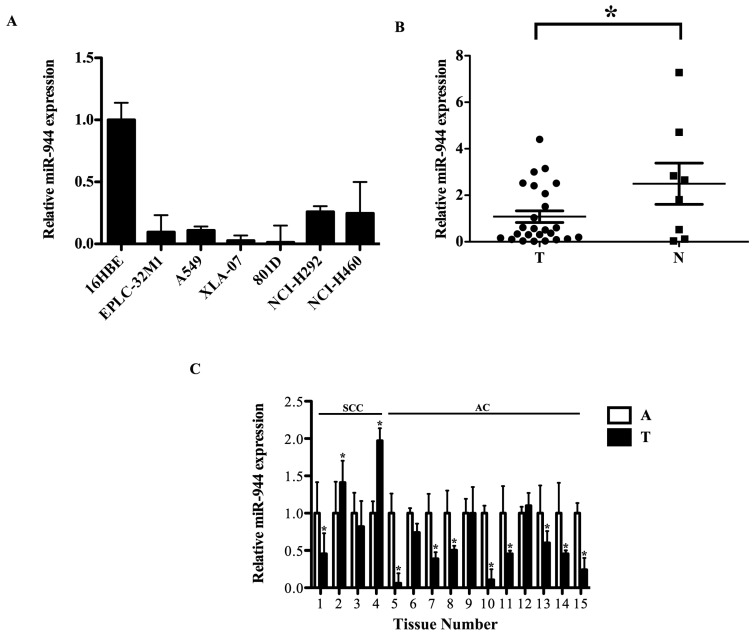
The reduced expression of miR-944 in NSCLC cell lines and specimens. (**A**) Relative expression levels of miR-944 in NSCLC cell lines compared with 16HBE; (**B**) Relative expression levels of miR-944 in NSCLC tissues and normal lung tissues; (**C**) Relative expression levels of miR-944 in paired NSCLC and adjacent non-tumor lung tissues. The significance was evaluated by *t*-test, * *p* < 0.05. (T: tumor; A: adjacent noncancerous tissues; SCC: squamous cell carcinoma; AC: adenocarcinoma; No. 1–4: SCC; No. 5–15: AC).

We performed qRT-PCR to verify expressions of miR-944. Consistent with the sequencing data, qRT-PCR assays showed that the expression level of miR-944 was markedly reduced in six NSCLC cell lines compared with 16HBE (Figure 2A). Furthermore, miR-944 expression was further determined in 25 NSCLC tissues and eight normal lung tissues. Compared with the eight normal lung tissues, the average level of miR-944 was decreased in the 25 NSCLC tissues (Figure 2B). Moreover, to reduce tumor heterogeneity, expressions of miR-944 were examined in 15 paired NSCLC samples. Compared with matched adjacent noncancerous tissues, miR-944 levels were significantly decreased in the 11 cases of NSCLC tissues (Figure 2C). These results indicated that the reduced expression of miR-944 was a frequent event in NSCLC cell lines and tissues, implying that miR-944 may be involved in lung carcinogenesis. We hereafter concentrated on the effects of miR-944 in tumorigenic processes of NSCLC.

### 2.3. The Overexpression of miR-944 Could Suppress the Proliferation of NSCLC Cells

**Figure 3:**
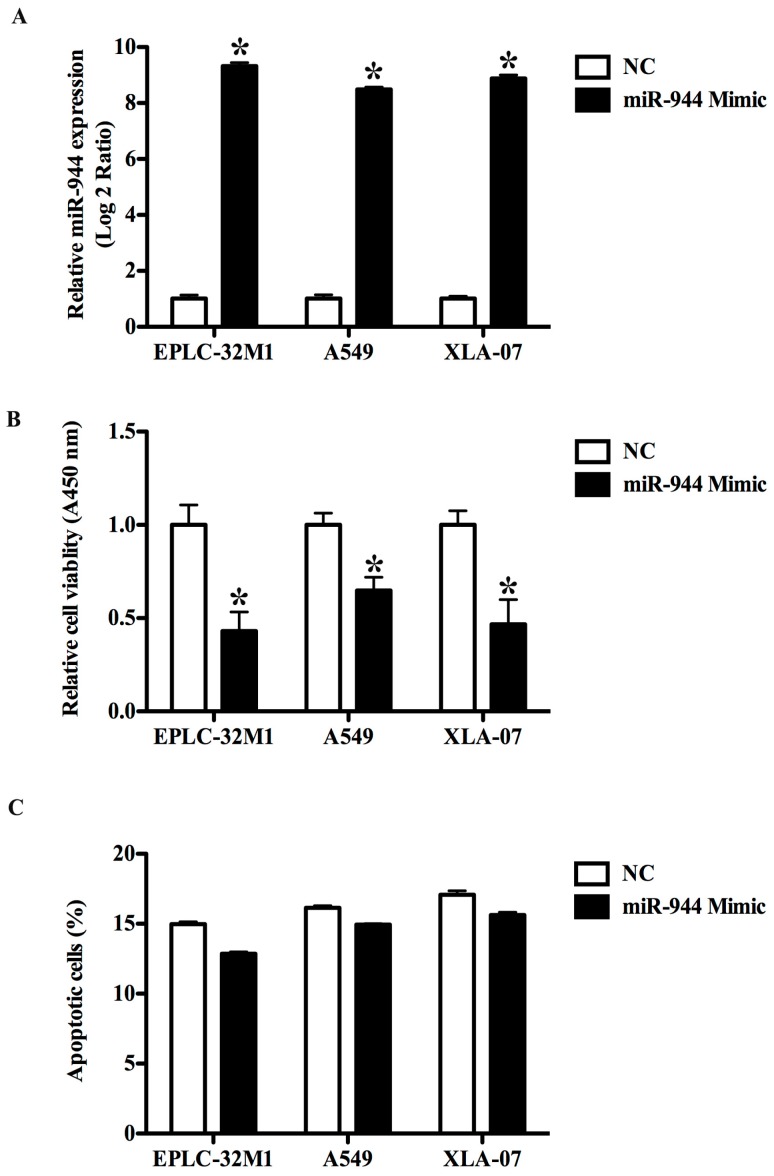
Effects of the ectopic miR-944 expression on the growth of cultured NSCLC cells. (**A**) Relative expression levels of miR-944 in EPLC-32M1, A549 and XLA-07 with or without miR-944 mimic transfection. The results of the cell proliferation assay (**B**) and apoptosis assay (**C**) in the three cell lines with or without miR-944 mimic transfection. The significance was evaluated by *t*-test, * *p* < 0.05.

To assess the effect of miR-944 on cell proliferation, we transfected EPLC-32M1, A549, and XLA-07 with miR-944 mimics and the negative control mimic. A qRT-PCR assay demonstrated that miR-944 expression was notably increased in the three NSCLC cell lines after miRNA mimic addition (Figure 3A). MTT assays showed that the enforced expression of miR-944 could significantly inhibit cell proliferation in EPLC-32M1, A549, and XLA-07 (Figure 3B). Apoptosis assays indicated that miR-944 overexpression had no significant effect on cell apoptosis (Figure 3C). Collectively, miR-944 overexpression could inhibit the proliferation of these tested NSCLC cells.

### 2.4. EPHA7 (a Target of miR-944) Was Screened in NSCLCs

To explore the mechanism by which miR-944 affects cell growth, we initially performed an mRNA expression profile in EPLC-32M1 cells with or without miR-944 mimic transfection. The array data revealed that the enforced overexpression of miR-944 induced 359 up-regulated and 346 down-regulated genes, respectively (Additional file 2). Next, we used Venny tool (http://bioinfogp.cnb.csic.es/tools/venny/index.html) to find the overlapped genes between predicted miR-944 targets and dyregulated mRNAs from the mRNA expression profile. The results showed that the overlapped genes included 30 up-regulated and 37 down-regulated genes from the mRNA expression profiling (Additional file 3). Furthermore, EPHA7 was selected for further investigation based on two strict criteria: (1) the chosen genes were associated with tumor initiation and progression, specifically in the context of lung cancer; (2) expression of the chosen genes inversely related to miR-944 expression.

### 2.5. The Expression Level of EPHA7 Is Inversely Correlated with the miR-944 Expression in NSCLC

**Figure 4:**
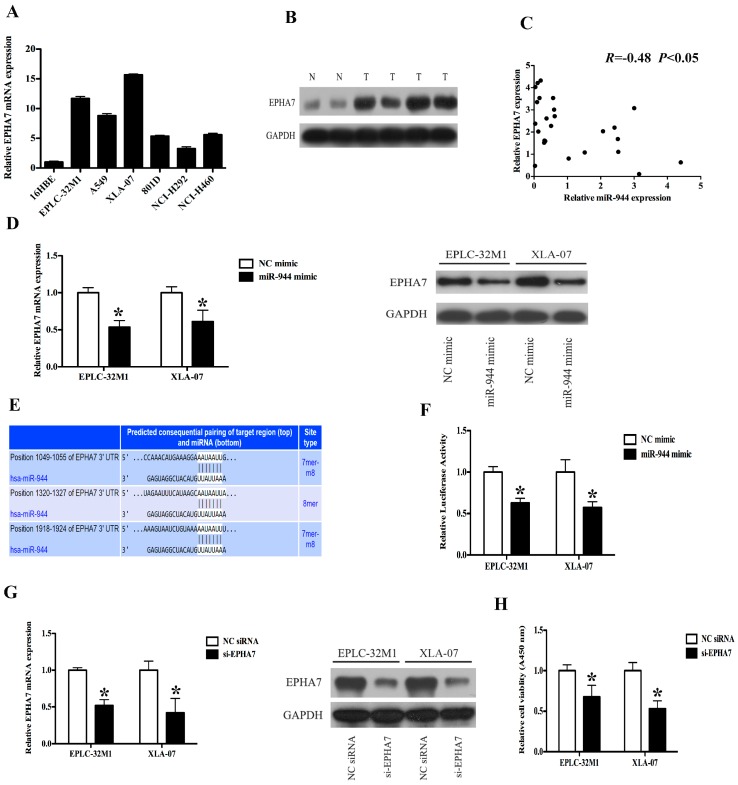
EPHA7 is a direct target of miR-944 in NSCLC. (**A**) The expressions of EPHA7 were examined by qRT-PCR in NSCLC cell lines; (**B**) The expressions of EPHA7 were examined by western blot in NSCLC tissues and noncancerous lung tissues; (**C**) The relationships between miR-944 and EPHA7 protein levels were analyzed using Pearson’s method in NSCLC tissues, *R* = −0.48, *p* < 0.05; (**D**) The mRNA and protein levels of EPHA7 were measured in EPLC-32M1 and XLA-07 cells transfected with the miR-944 mimic and negative control (NC); (**E**) Sequences of the putative miR-944 binding sites; (**F**) The relative luciferase activity was determined using luciferase reporters with EPHA7-3′UTR in EPLC-32M1 and A549 cells transfected with the miR-944 mimic and negative control (NC); (**G**) The EPHA7 expression was analyzed using qRT-PCR and western blot in EPLC-32M1 and XLA-07 cells transfected with the siRNA (si-EPHA7) and negative control (NC); (**H**) The proliferation rates were analyzed using proliferation assay in EPLC-32M1 and XLA-07 cells after transfection with the siRNA (si-EPHA7) and negative control (NC). GAPDH was used as an internal control (**B**,**D**,**G**). The significance was evaluated by *t*-test (**A**,**B**,**D**,**F**–**H**), * *p* < 0.05.

Through qRT-PCR, we found that EPHA7 expression levels were relatively higher in the NSCLC cell lines whereas the miR-944 levels were relatively lower in these cell lines (Figure 2A and Figure 4A). To further explore the relationship between miR-944 and EPHA7 expression, we assessed EPHA7 expression in a cohort of 25 NSCLC tissues and eight noncancerous lung tissues. The data showed that levels of EPHA7 protein were remarkably elevated in a large part of NSCLC samples compared with the noncancerous samples (Figure 4B, Additional file 4) and appeared to be inversely correlated with miR-944 expression in NSCLC tissues (Figure 4C). In 15 paired tissues, western blot analysis confirmed the overexpression of EPHA7 in tumor tissues compared with matched adjacent noncancerous tissues (Additional file 5A), and a correlation assay demonstrated the inverse correlation between EPHA7 protein expression and miR-944 expression, especially in AC tissues (Additional file 5B,C). Thus, the inverse expression of EPHA7 and miR-944 indicated that EPHA7 might be regulated by miR-944 in NSCLC cells.

### 2.6. The Up-Regulation of miR-944 Could Suppress the EPHA7 Expression

To determine whether miR-944 could regulate the EPHA7 expression, we firstly transfected miR-944 mimic and its control into EPLC-32M1 and XLA-07. After 72 hours of miRNA mimic transfection, qRT-PCR was employed to measure the miR-944 level (Additional file 6). Subsequently, we examined levels of EPHA7 mRNA and protein in these cells. As predicted, the forced expression of miR-944 significantly suppressed levels of EPHA7 protein (Figure 4D).

### 2.7. miR-944 Directly Targeted EPHA7 by Interacting with Its 3′-Untranslated Regions (UTRs)

To verify whether miR-944 is directly interacting with EPHA7, we cloned 3′-UTR fragments of EPHA7, which included the predicted miR-944 binding sites (Figure 4E), into luciferase reporter construct. We found that the co-transfection of miR-944 could significantly reduce the relative luciferase activities of the reporter with EPHA7-3′-UTR (Figure 4F). Altogether, these data imply that miR-944 may attenuate the EPHA7 expression by directly targeting its 3′-UTR.

### 2.8. The Down-Regulation of EPHA7 Could Suppress the Proliferation of NSCLC Cells

We reduced the endogenous EPHA7 expression using specific small interfering RNA (siRNA) in EPLC-32M1 and XLA-07 cells and further investigated whether EPHA7 could promote cell growth in NSCLC. As shown in Figure 4G, EPHA7 was notably downregulated at mRNA and protein levels by siRNAs. A cell proliferation assay showed that depletion of EPHA7 could obviously suppress the proliferation of NSCLC cells (Figure 4H), indicating that EPHA7 could promote the proliferation of NSCLC cells.

## 3. Discussion

In recent years, multiple studies have indicated that deregulated miRNAs play crucial roles in the development and progression of cancer, so identification of cancer-related miRNAs and their target genes involved in carcinogenesis attracted particular attention. Deep-sequencing technology is a robust tool to determine expression of all annotated miRNAs and discover novel miRNAs [8]. Next generation sequencing has been employed to identify differentially expressed miRNAs in NSCLC tissues [9]. Here, we utilized cultured NSCLC cells for small RNA deep sequencing. Consistent with previous reports [10,11], miR-10b-5p (miR-10b) and miR-224 were overexpressed, miR-200c-3p (miR-200c) and miR-141-3p (miR-141) were significantly reduced in NSCLC cell lines, and our laboratory also reported reduced expression of miR-129-1-3p and miR-203a in the NSCLC sample [12,13]. Meanwhile, we also revealed several novel miRNAs with so far unknown biological functions in lung tumorigenesis, such as miR-934, miR-935 and miR-936. In the present study, given the high score across algorithms and the number of the target mRNAs, miR-944 was chosen for further investigation.

Several studies demonstrated that miR-944 high-expression was associated with better prognosis and chemotherapeutic sensitivity in human cancers including bladder [14], pancreatic [15] and colorectal [16]. In this study, we found down-regulation of miR-944 in NSCLCs through deep-sequencing of miRNAs using cultured cells. Furthermore, to verify the sequencing data, we examined the expression of miR-944 in NSCLC cell lines and clinical NSCLC specimens using qRT-PCR and found that miR-944 was downregulated in the majority of NSCLC cell lines and tissues used in this study. Cell functional assays showed that miR-944 overexpression could suppress cell proliferation in EPLC-32M1, A549 and XLA-07, but has little effect on cell apoptosis and cycle progression, indicating that miR-944 contributes to the proliferation of NSCLC cells. However, we also observed that miR-944 was not downregulated in some NSCLC tissue, even miR-944 showed the up-regulation in the minority of NSCLC tissue. Moreover, we have noticed that miR-944 displayed distinctive expression between lung SCC and AC. These observations reflected that: (1) NSCLC cells possess the heterogeneity and diversity of miR-944 regulation; (2) the regulation of miR-944 is very complicated and varied in different subtypes of NSCLC. In a previous study, miR-944 functioned as an oncogene in lung SCC by promoting cell proliferation, migration and invasion in a previous study [17]. Our results were inconsistent with the previous studies about miR-944 in lung cancer; one possible reason may be that the majority of NSCLC tissues were lung AC in our research. In line with previous studies, the host gene encoding miR-944 (*TP63*, also named as p40 and ΔNp63) is considered as a differentiation marker of the squamous cell, that can distinguish SCC from AC [18,19]. It was well accepted that one gene expression might be distinct in different cellular contexts, which may result in complex functional effects including cellular proliferation, apoptosis, drug resistance, migration and invasion. Therefore, miR-944 might have different roles between lung SCC and AC. However, further studies are needed to determine the roles and molecular mechanism of miR-944 between SCC and AC in human NSCLC.

To explore the mechanism by which miR-944 affects cell proliferation, we combined in silico target prediction with mRNA expression profiling in EPLC-32M1 (SCC) cells with miR-944 overexpression, which was a well-established approach to identify miRNA targets regulated at the transcriptional level [20]. Results from the integrated analysis showed that miR-944 may potentially interact with the 3’-UTR of many genes. Of these target genes, the obvious one is EPHA7, which was reported to be up-regulated and, significantly, positively associated with the proliferation of lung cancer cells [21]. EPHA7, a member of the EPHA family, belongs to receptor kinases, and plays diverse roles in carcinogenesis. EPHA7 acted as a TSG in modulating cell growth in human colorectal [22], gastric [23], and prostate carcinomas [24]. The truncated form of EPHA7 (EPHA7^TR^) was revealed as a soluble tumor suppressor for follicular lymphoma by inhibiting EPHA2 signaling [25], which makes EPHA7 a promising targeted therapeutic polypeptide. However, other reports showed that EPHA7 has a promoting role in human lung cancers, laryngeal carcinomas, and glioblastoma like an oncogene [21,26,27], but detailed mechanisms are not clear so far. They indicated that EPHA7 might be able to exert tissue specific or cell specific functions. Therefore, it was valuable to disclose the molecular mechanism underlying the process of EPHA7 overexpression in NSCLC. Here, we used siRNA to knockdown EPHA7 expression and further confirmed that the silencing of EPHA7 could suppress the growth of NSCLC cells. Moreover, EPHA7 is overexpressed and inversely associated with the miR-944 expression in both cultured NSCLC cells and NSCLC tissues (NSCLC tissues vs. normal lung tissues; NSCLC tissues vs. their adjacent noncancerous lung tissues within the same patient), which was consistent with the general inverse relationship between the expression level of miRNAs and the targets. Furthermore, transfection of miR-944 mimics could reduce EPHA7 levels. Luciferase reporter assays also revealed that miR-944 bound to the 3′-UTR of EPHA7. Our findings indicated that EPHA7 is the direct target of miR-944 and provides the first evidence to explain EPHA7 overexpression in NSCLC that may result from down-regulation of miR-944. However, the correlation coefficient is only −0.48 between miR-944 and EPHA7 in NSCLC tissues; in the paired NSCLC tissues, the correlation coefficient is −0.60. These results indicated that: (1) the EPHA7 expression is not associated with miR-944 in the minority of NSCLC, although the EPHA7 expression might be inversely regulated by miR-944 in the majority of NSCLC tissue; (2) apart from miR-944, EPHA7 regulation is involved in other mechanisms. Interestingly, the correlation coefficient reached −0.65 in the paired AC tissues. We speculated that in most lung AC, the EPHA7 expression was regulated by miR-944. In a previous study, we suggested that there are regulatory relationships between oncogenes and TSGs apart from their functional synergy; the oncogene–miRNA–TSG networks are one of the mechanisms for the regulatory relationships, and the networks may play roles in carcinogenesis [28]. Here, we suggested that the EPHA7 over-expression was regulated by means of, but not limited to, miR-944 down-regulation in NSCLC, and then up-regulation of EPHA7 which promoted the cell growth and participated in the development and progression of cancer.

## 4. Experimental Section

### 4.1. Human Tissue Specimens and Cell Lines

NSCLC and adjacent non-tumor lung tissues were collected from local hospitals. No patients were treated before undergoing surgery. Furthermore, lung tissues, obtained from patients with lung bullous and inflammatory pseudotumor through surgical operation, were used as “lung normal tissues”. This study was approved by the Ethics Committee for Human Medicine Research, Kunming Institute of Zoology, Chinese Academy of Sciences. The clinical characteristics of patients are listed in Additional file 7.

NSCLC cell lines EPLC-32M1 (SCC), A549 (AC), 801D (LCC), NCI-H292 (mucoepidermoid carcinoma), NCI-H460 (LCC), 16HBE (immortalized human bronchial epithelial cells), and PT-67 (retrovirus packing cell) have been described previously [12]. XLA-07 (AC) was gifted by Ma, L.J. [29]. All cell lines were maintained in RPMI 1640 or Dulbecco’s modified Eagle’s medium containing 10% fetal bovine serum (FBS) and antibiotics at 37 °C in 5% CO_2_.

### 4.2. miRNA Expression Profiling

Total RNA was isolated using the Trizol reagent (Sigma-Aldrich, St. Louis, MO, USA). miRNA expression profiling was determined in EPLC-32M1, A549, 801D, and 16HBE through small RNA deep sequencing using Hiseq 2000 platform by BGI Tech (Shenzhen, China; http://bgitechsolutions.com).

### 4.3. miRNA Target Prediction

miRNA targets were predicted using miRwalk, Diana-microT, miRanda, miRDB, RNAbybrid, PICTAR4/5, PITA, RNA22 and TargetScan. Predicted targets were identified by at least five of these programs. To obtain the miRNA/target network, candidate miRNAs and corresponding predicted targets were submitted to Cytoscape (version: 3.1.1). According to the miRNA degree, the network of miRNA-mRNA interaction was established.

### 4.4. Quantitative Real-Time PCR (qRT-PCR)

Total RNAs and miRNAs from NSCLC cell lines and tissues were isolated using TRIzol reagent (Sigma-Aldrich). The expression of miR-944 was analyzed with the miScript system (QIAGEN, Hilden, Germany) according to the manufacturer’s instructions. The qRT-PCR assays of mRNA and miRNA expression levels were performed as previously described [12,28]. The housekeeping genes GAPDH and U6 snRNA were used as internal controls for the mRNA and miRNA assays, respectively. The primers used are shown in Additional file 8.

### 4.5. Oligonucleotide Transfection

miR-944 mimics and its cognate controls were purchased from RiBoBio company (Guangzhou, China). The EPHA7 siRNA and control siRNA were obtained from Santa Cruz Biotechnology (Santa Cruz, CA, USA). The transfection was performed using Lipofectamine 2000 (Invitrogen, Carlsbad, CA, USA) following the manufacturer’s instructions.

### 4.6. MRNA Expression Profiling

Determination of mRNA profiling was performed in EPLC-32M1 cells treated with and without miR-944 mimic using Agilent 60K Human Gene Expression array by CapitalBio Corporation (Beijing, China; http://www.capitalbio.com).

### 4.7. Western Blot

Cells were harvested and lysed in RIPA buffer (Beyotime Biotechnology, Shanghai, China). Cell lysates were resolved by 8% sodium dodecylsulfate polyacrylamide gel electrophoresis (SDS-PAGE) and transferred onto PVDF membranes (Millipore, Bedford, MA, USA). The membrane was incubated with EPHA7 antibody (sc-1015; Santa Cruz Biotechnology, Santa Cruz, CA, USA), or GAPDH antibody (G8795; Sigma-Aldrich, St. Louis, MO, USA).

### 4.8. Cell Growth and Apoptosis Assay

Cell functional assays were performed as previously described [12]. The cell proliferation assay was based on 3-(4,5-dimethylthiazol-2-yl)-2,5-Diphenyltetrazolium bromide (MTT) methods. After transfection, cells were seeded in 96-well plates and cell viability was assessed. The cell apoptosis assay was assessed using the Fluorescein Isothiocyanate (FITC)-labeled Annexin V Kit (BD Pharmingen, San Diego, CA, USA) according to the manufacturer’s instructions. Briefly, cells were stained with annexin V-FITC and propidium iodide (PI) and then were analyzed on a FACScan flow cytometer (Becton Dickinson, Mountain View, CA, USA).

### 4.9. Luciferase Reporter Assays

For generation of EPHA7 luciferase reporter plasmid (pGL-EPHA7), the 3′-untranslated region (3′-UTR) containing the miR-944 binding site was cloned between the *NheI* and *BglII* restriction sites of the pGL-3 basic vector (Promega, Madison, WI, USA) using a PCR-amplified fragment. The primers used are listed in Additional file 8.

Each reporter construct and the *Renilla* luciferase expression plasmid (pRL-TK) were co-transfected into cultured cells with the miR-944 mimic or its negative control using Lipofectamine 2000 (Invitrogen, Carlsbad, CA, USA). Forty-eight hours after transfection, the levels of luciferase activity were identified using the Dual-Luciferase Reporter System (Promega, Madison, WI, USA) according to the manufacturer’s instructions. The pRL-TK plasmid was used as an internal control.

### 4.10. Statistical Analysis

All the data are presented as the mean ± standard deviation (SD) from at least three independent experiments. The statistical significant was calculated using the Student’s *t*-test. The relationships between the miR-944 and EPHA7 expression was determined by Pearson’s method. SPSS 17.0 software package (Chicago, IL, USA) was used for all the statistical analysis. The level of statistical significance was set at 0.05 for all the tests.

## 5. Conclusions

We identified that miR-944 was frequently down-regulated in human lung cancer. In vitro, the overexpression of miR-944 was able to effectively suppress proliferation of the NSCLC cell. We also disclosed that EPHA7 was the direct target of miR-944, and down-regulation of EPHA7 caused by overexpression of miR-944 could suppress the proliferation of NSCLC cells. Thus, the identification of the miR-944–EPHA7 pathway may provide potential clues for understanding the molecular mechanism underlying NSCLC and the treatment of this fatal disease.

## Figures and Tables

**Table 1:** Most dyregulated microRNAs (miRNAs) in non-small cell lung cancer (NSCLC) cells compared with the normal cell 16HBE. (Log_2_(Fold change) > 3, *p* < 0.001).

miRNA	Log_2_(Fold Change)			Cytoband
	EPLC-32M1 vs. 16HBE	801D vs. 16HBE	A549 vs. 16HBE	
**Up-Regulated miRNAs**				
hsa-miR-10a-3p	4.446	6.715	4.289	17q21.32
hsa-miR-10a-5p	5.816	8.581	4.626	17q21.32
hsa-miR-10b-5p	8.912	6.686	8.627	17q21.32
hsa-miR-137	8.775	9.571	8.524	1p22
hsa-miR-140-3p	4.144	4.659	3.415	16q22.1
hsa-miR-193a-5p	4.103	6.348	4.054	17q11.2
hsa-miR-224-5p	13.982	9.493	12.369	Xq28
hsa-miR-2682-5p	8.822	10.672	8.441	Chromosome1: (98510798-98510907)
hsa-miR-31-3p	3.050	4.505	5.289	9p21.3
hsa-miR-3179	7.568	8.893	7.469	16p13.11
hsa-miR-320d	5.557	3.675	3.404	13q14.11
hsa-miR-365b-5p	5.031	7.127	6.003	17q11.2
hsa-miR-452-5p	13.175	7.857	10.770	Xq28
hsa-miR-935	7.900	10.634	7.664	19q13.42
**Down-Regulated miRNAs**				
hsa-miR-129-1-3p	9.110	3.802	6.340	7q32
hsa-miR-141-3p	6.884	11.752	7.398	12p13.31
hsa-miR-200c-3p	7.068	9.662	7.691	12p13.31
hsa-miR-203a	4.007	3.724	9.962	14q32.33
hsa-miR-205-3p	6.766	6.766	6.766	1q32.2
hsa-miR-544b	6.650	6.650	6.6501	Chromosome3: (124451286-124451363)
hsa-miR-934	8.313	8.313	8.313	Xq26.3
hsa-miR-936	7.650	7.650	7.650	10q25.1
hsa-miR-944	3.597	7.235	3.4975	3q28

Log_2_(Fold change) (NSCLC cells vs. 16HBE) was used to evaluate the differentially expressed miRNA between NSCLC cell lines and 16HBE.

## Supplementary Materials

Supplementary materials can be found at www.mdpi.com/1422-0067/17/9/1493/s1. Additional file 1. Dyregulated miRNAs in NSCLC cell lines compared with immortalized human bronchial epithelial cell line (16HBE). Additional file 2. The differently expressed genes in EPLC-32M1 cells with or without miR-944 mimic transfection using microarray analysis. Additional file 3. The differentially expressed genes selected through the integrated analysis of miR-944 mediated-mRNA expression profiles with its predicted targets. Additional file 4. EPHA7 expression level in NSCLC tissues and normal lung tissues. (N: normal lung tissues, T: tumor tissues). Additional file 5. EPHA7 expression levels in the paired NSCLC tissues. (A) The expressions of EPHA7 were examined by western blot in the paired NSCLC tissues; (B) The co-relation of miR-944 and EPHA7 protein levels were analyzed using Pearson’s method in the paired NSCLC tissues, *R* = −0.60, *p* < 0.05; (C) The co-relation of miR-944 and EPHA7 protein levels were analyzed using Pearson’s method in the paired lung AC tissues, *R* = −0.65, *p* < 0.05. A: adjacent noncancerous tissues. T: tumor. No. 1–4: squamous cell carcinoma (SCC). No. 5–15: adenocarcinoma (AC). Additional file 6. miR-944 expression levels after 72 hours of miRNA mimic transfection. Additional file 7. Clinical and pathologic information of the patients. Additional file 8. The primers used in this study.
