# Supplementary material for: MicroRNA-944 Affects Cell Growth by Targeting EPHA7 in Non-Small Cell Lung Cancer
Source: Int J Mol Sci. 2016 Sep 26;17(10):1493. doi: 10.3390/ijms17101493 (PMC5085614; doi:10.3390/ijms17101493)
Supplement: Supplementary file 1 [file ijms-17-01493-s001.zip › Additional file 7.pdf]

**Additional file 7A.** Clinical and pathologic information of patients.

| Case No. | Gender | Age | Smoking | Histological Type | TNM | Stage |
|---|---|---|---|---|---|---|
| **1** | Male | 42 | YES | AC | T2N1M0 | IIB |
| **2** | Male | 51 | YES | AC | T2N0M0 | IB |
| **3** | Female | 56 | NO | AC | T2N1M0 | IIB |
| **4** | Male | 39 | NO | SCC | T2N0M0 | IB |
| **5** | Female | 66 | NO | AC | T1N0M0 | IA |
| **6** | Male | 76 | YES | SCC | T2N0M0 | IB |
| **7** | Female | 42 | NO | AC | T0M0N0 | IA |
| **8** | Female | 56 | NO | AC | T2N0M0 | IB |
| **9** | Female | 58 | NO | AC | T2N1M0 | IIB |
| **10** | Male | 44 | YES | AC | T1N0M0 | IA |
| **11** | Female | 62 | NO | AC | T2N0M0 | IB |
| **12** | Male | 39 | YES | AC | T1N1M0 | IIA |
| **13** | Female | 57 | YES | AC | T2N2M0 | IIIA |
| **14** | Male | 43 | YES | AC | T2N1M0 | IIB |
| **15** | Male | 70 | YES | SCC | T2N0M0 | IB |
| **16** | Male | 66 | NO | SCC | T2N0M0 | IB |
| **17** | Male | 62 | YES | AC | T1N1M0 | IIA |
| **18** | Male | 69 | YES | AC | T0M0N0 | IA |
| **19** | Male | 69 | YES | AC | T2N0M0 | IB |
| **20** | Female | 50 | NO | AC | T2N0M0 | IB |
| **21** | Female | 37 | NO | AC | T2N0M0 | IB |
| **22** | Female | 52 | NO | AC | T2N0M0 | IB |
| **23** | Male | 61 | YES | AC | T2N1M0 | IIB |
| **24** | Female | 40 | NO | AC | T2N0M0 | IB |
| **25** | Male | 67 | YES | SCC | T1N0M0 | IA |
| **26** | Female | 49 | NO | LB | None | None |
| **27** | Male | 58 | YES | IP | None | None |
| **28** | Male | 57 | YES | LB | None | None |
| **29** | Male | 56 | YES | IP | None | None |
| **30** | Male | 47 | YES | IP | None | None |
| **31** | Male | 64 | NO | IP | None | None |
| **32** | Male | 61 | YES | IP | None | None |
| **33** | Male | 70 | YES | IP | None | None |

AC, adenocarcinomas; SCC, squamous cell carcinomas; IP, inflammatory pseudotumor; LB, lung bullous; No. 1–25, NSCLC patients; No. 26–33, patients with lung bullous or inflammatory pseudotumor.

**Additional file 7B.** Clinical and pathologic information of lung cancer patients (paired cancer and match normal tissues).

| Case No. | Gender | Age | Smoking | Histological Type | TNM | Stage |
|---|---|---|---|---|---|---|
| **1** | M | 63 | NO | SCC | T2N0M0 | IB |
| **2** | M | 43 | YES | SCC | T2N1M0 | IIB |
| **3** | M | 55 | NO | SCC | T2N2M0 | IIIA |
| **4** | FM | 69 | NO | SCC | T2N2M0 | IIIA |
| **5** | FM | 60 | NO | AC | T2N0M0 | IB |
| **6** | FM | 53 | NO | AC | T2N0M0 | IB |
| **7** | FM | 39 | NO | AC | T2N0M0 | IB |
| **8** | FM | 42 | NO | AC | T0N0M0 | IA |
| **9** | M | 44 | YES | AC | T1N0M0 | IA |
| **10** | FM | 55 | NO | AC | T0N0M0 | IA |
| **11** | FM | 36 | NO | AC | T2N0M0 | IB |
| **12** | FM | 60 | NO | AC | T2N0M0 | IB |
| **13** | M | 49 | YES | AC | T2N0M0 | IB |
| **14** | FM | 48 | NO | AC | T2N0M0 | IB |
| **15** | FM | 50 | NO | AC | T2N0M0 | IB |

M, male; F, female; SCC, squamous cell carcinomas; AC, adenocarcinomas.
