# Supplementary material for: MicroRNA-944 Affects Cell Growth by Targeting EPHA7 in Non-Small Cell Lung Cancer
Source: Int J Mol Sci. 2016 Sep 26;17(10):1493. doi: 10.3390/ijms17101493 (PMC5085614; doi:10.3390/ijms17101493)
Supplement: Supplementary file 1 [file ijms-17-01493-s001.zip › Additional file 8.pdf]

Additional file 8. The primers used in this study.

**Plasmid Construction**	**Forward Primers (5′–3′); Reverse Primers (5′–3′)**
EPHA7 3′-UTR	CTAGCTAGCTCGGATCCATACATACTATAAGGCACT;GAAGATCTGGAAAGACACCGTTTGCATG
**QRT-PCR Analysis**	**Forward Primers (5′–3′); Reverse Primers (5′–3′)**
EPHA7	GTGAAGATGGGTATTACAGGGC; CAACTGCACCGCTTACACAAT
GAPDH	TGTTGCCATCAATGACCCCTT; CTCCACGACGTACTCAGCG

For miRNAs expression analysis, the forward primers of miR-944 were purchased from Guangzhou RiboBio Co., Ltd；the U6 and miRNA reverse primers were purchased from Qiagen, Hilden, Germany.
