# Supplementary material for: MicroRNA-944 Affects Cell Growth by Targeting EPHA7 in Non-Small Cell Lung Cancer
Source: Int J Mol Sci. 2016 Sep 26;17(10):1493. doi: 10.3390/ijms17101493 (PMC5085614; doi:10.3390/ijms17101493)
Supplement: Supplementary file 1 [file ijms-17-01493-s001.zip › Additional file 3.pdf]

Additional file 3. The differentially expressed genes selected through the integrated analysis of miR944 mediated-mRNA expression profiles with its predicted targets.

Gene Symbol	GenBank	Gene Name	Fold Change	*p*
**Down-regulated genes**				
FUT9	NM_006581	fucosyltransferase 9	−13.7978	*
AP1S3	NM_001039569	adaptor-related protein complex 1, sigma 3 subunit,	−2.3475	*
ZPLD1	NM_175056	zona pellucida-like domain containing 1	−2.1427	*
DBT	NM_001918	dihydrolipoamide branched chain transacylase E2	−2.0771	*
TAPT1	NM_153365	transmembrane anterior posterior transformation 1	−3.0689	*
EPHA7	NM_004440	EPH receptor A7	−22.0652	*
MS4A2	NM_000139	membrane-spanning 4-domains, subfamily A, member 2	−6.0955	*
PAN3	NM_175854	poly(A) specific ribonuclease subunit homolog	−11.7924	*
PCLO	NM_033026	piccolo	−14.2148	*
HLA-F	NM_018950	major histocompatibility complex, class I, F	−2.3446	*
MMP13	NM_002427	matrix metallopeptidase 13	−5.6270	*
RNF20	NM_019592	ring finger protein 20	−2.0615	*
KCTD16	NM_020768	potassium channel tetramerisation domain containing 16	−2.1983	*
CCDC15	NM_025004	coiled-coil domain containing 15	−5.0194	*
ZNF605	NM_183238	zinc finger protein 605	−4.1594	*
CDKN2B	NM_004936	cyclin-dependent kinase inhibitor 2B	−2.3499	*
PASD1	NM_173493	PAS domain containing 1	−2.1243	*
AMOT	NM_001113490	angiomotin	−4.1110	*
CYP7A1	NM_000780	cytochrome P450, family 7, subfamily A, polypeptide 1	−2.5574	*
F5	NM_000130	coagulation factor V	−5.3073	*
SP8	NM_198956	Sp8 transcription factor	−15.9073	*
GNAO1	NM_138736	guanine nucleotide binding protein,<br>alpha activating activity polypeptide O	−2.3351	*
GUCA1B	NM_002098	guanylate cyclase activator 1B	−3.7807	*
BCL11A	NM_018014	B-cell CLL/lymphoma 11A (zinc finger protein)	−6.9443	
AGPAT4	NM_020133	1-acylglycerol-3-phosphate O-acyltransferase 4	−2.0476	*
PLXDC1	NM_020405	plexin domain containing 1	−2.3056	*
PAK7	NM_020341	p21 protein (Cdc42/Rac)-activated kinase 2	−30.6842	*
MPP5	NM_022474	membrane protein, palmitoylated 5	−2.1422	*
SLC7A4	NM_004173	solute carrier family 7, member 4	−2.9090	*
ZNF215	NM_013250	zinc finger protein 215	−12.9707	*
PRRG3	NM_024082	proline rich Gla 3	−2.8356	*
SSH2	NM_001282130	slingshot protein phosphatase 2	−2.5703	*
GCNT3	NM_004751	glucosaminyl (N-acetyl) transferase 3, mucin type	−2.3665	*
PIGM	NM_145167	phosphatidylinositol glycan anchor biosynthesis, class M	−2.0519	*
STXBP5L	NM_014980	syntaxin binding protein 5-like	−3.1841	*
SLC23A2	NM_203327	solute carrier family 23, member 2	−2.0665	*
MORC1	NM_014429	MORC family CW-type zinc finger 1	−7.6522	*

Additional file 3. *Cont.*

Gene Symbol	GenBank	Gene Name	Fold Change	*p*
**Up-regulated genes**				
MIER3	NM_152622	mesoderm induction early response 1, family member 3	4.0154	*
RTKN2	NM_145307	rhotekin 2	3.1024	*
GRM8	NM_000845	glutamate receptor, metabotropic 8	6.6306	*
IL8	NM_000584	interleukin 8	16.7740	*
BRWD1	NM_018963	bromodomain and WD repeat domain containing 1	2.6391	*
BNC2	NM_017637	basonuclin 2	2.0136	*
TLE4	NM_001083962	transcription factor 4	6.3657	*
ARHGAP26	NM_004815	Rho GTPase activating protein 26	11.2820	*
TOMM70A	NM_014820	translocase of outer mitochondrial membrane 70 homolog A (S. cerevisiae)	2.2392	*
STAG2		stromal antigen 2	2.5660	*
RAPGEF4	NM_007023	Rap guanine nucleotide exchange factor (GEF) 4	2.521	*
ZNF618	NM_133374	zinc finger protein 618	2.1561	*
UPP2	NM_173355	uridine phosphorylase 2	21.5104	*
DPYD	NM_001160301	dihydropyrimidine dehydrogenase	2.3433	*
ECM2		extracellular matrix protein 2, female organ and adipocyte specific	6.9829	*
DISC1	NM_001164549	disrupted in schizophrenia 1	2.3467	*
BBS9	NM_014451	Bardet-Biedl syndrome 9	3.4804	*
GUCA1B	NM_002098	guanylate cyclase activator 1B	6.2035	*
ITK	NM_005546	IL2-inducible T-cell kinase	9.0695	*
FOXJ2	NM_018416	forkhead box J2	2.0712	*
KLHL14	NM_020805	kelch-like 14 (Drosophila)	3.8543	*
SCN1A	NM_006920	sodium channel, voltage-gated, type I, alpha subunit	11.5761	*
IQCH	NM_022784	IQ motif containing H	7.4185	*
TEC	NM_003215	tec protein tyrosine kinase	3.2338	*
TRPM8	NM_024080	transient receptor potential cation channel, subfamily M, member 8	4.1876	*
MLPH	NM_024101	melanophilin	5.0901	*
ZNF750	NM_024702	zinc finger protein 750	12.5551	*
VASH2	NM_024749	vasohibin 2	2.5064	*
AXIN2	NM_004655	axin 2	2.0091	*
LRAT	NM_004744	lecithin retinol acyltransferase	5.8564	*

* $p < 0.05$.
