# Supplementary figures and images for: MicroRNA-944 Affects Cell Growth by Targeting EPHA7 in Non-Small Cell Lung Cancer
Source: Int J Mol Sci. 2016 Sep 26;17(10):1493. doi: 10.3390/ijms17101493 (PMC5085614; doi:10.3390/ijms17101493)

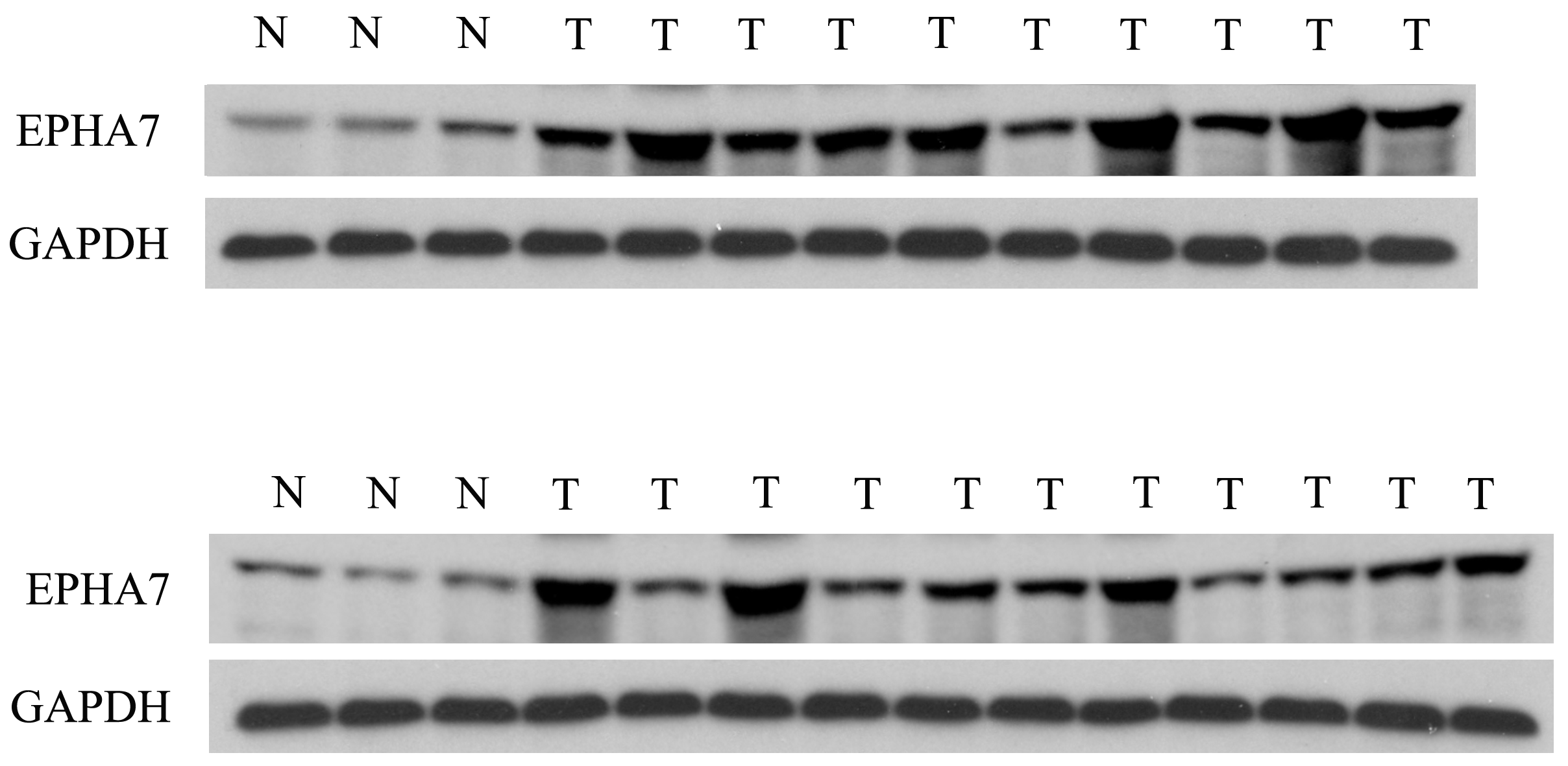

Supplement: Supplementary file 1 [file ijms-17-01493-s001.zip › Additional file 4.tif]

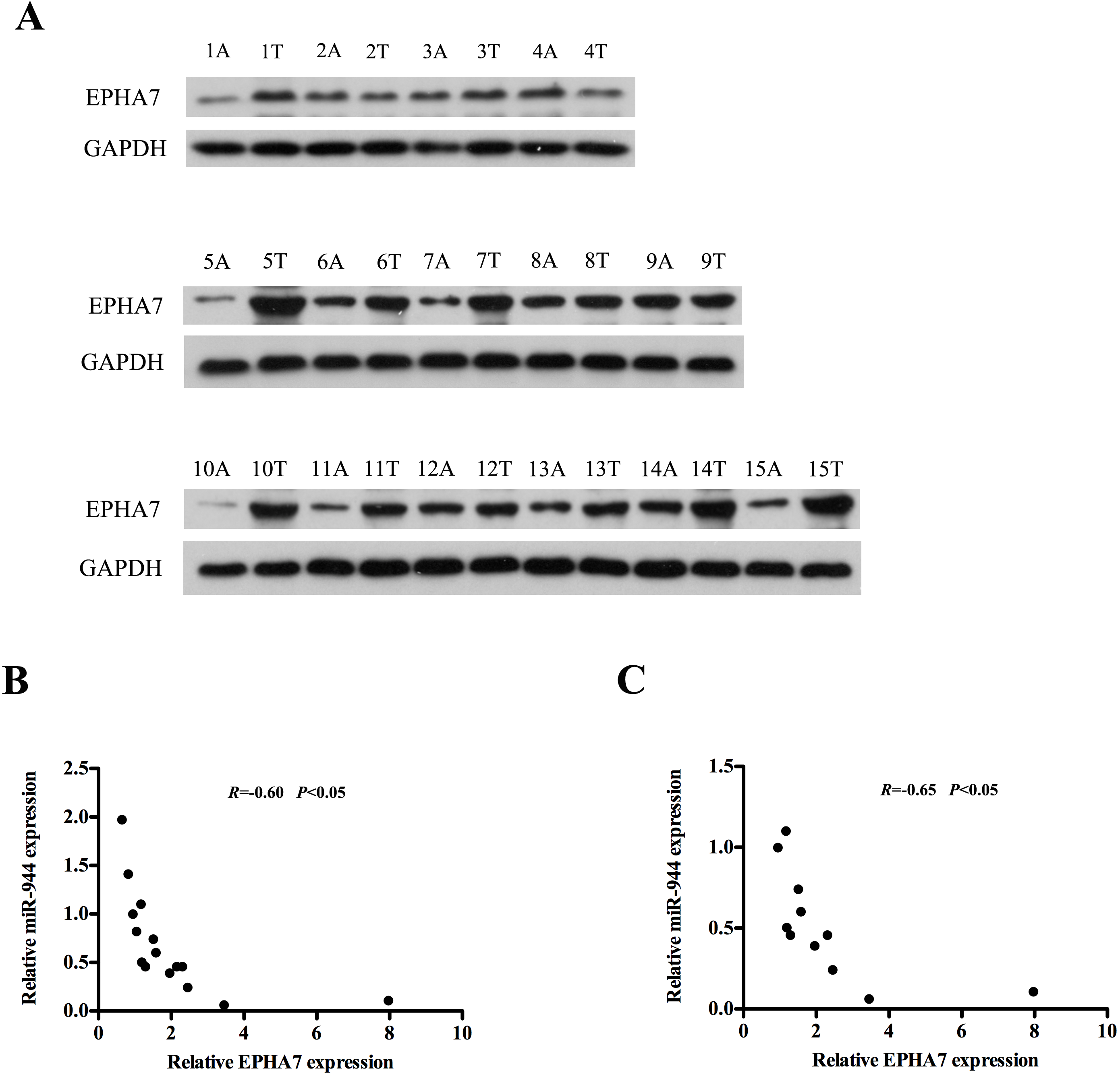

Supplement: Supplementary file 1 [file ijms-17-01493-s001.zip › Additional file 5.tif]

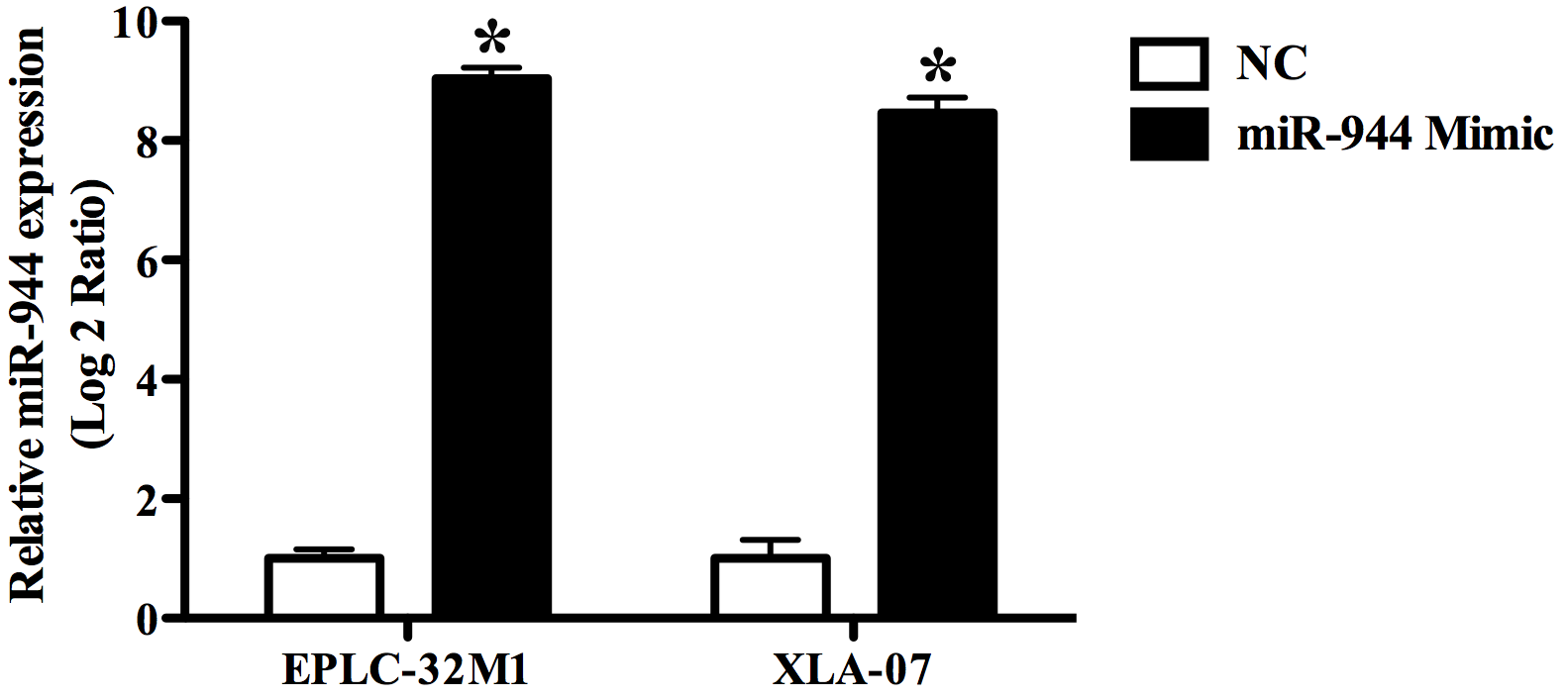

Supplement: Supplementary file 1 [file ijms-17-01493-s001.zip › Additional file 6.tif]
